# The fracture predictive ability of a musculoskeletal composite score in old men – data from the MrOs Sweden study

**DOI:** 10.1186/s12877-019-1106-2

**Published:** 2019-03-22

**Authors:** Felix Cronholm, Björn E. Rosengren, Jan-Åke Nilsson, Claes Ohlsson, Dan Mellström, Eva Ribom, Magnus K. Karlsson

**Affiliations:** 1 Clinical and Molecular Osteoporosis Research Unit, Department of Clinical Sciences and Orthopedics, Lund University, Skåne University Hospital, Malmö, Sweden; 2 Centre for Bone and Arthritis Research, Department of Internal Medicine and Clinical Nutrition, Institute of Medicine, Sahlgrenska Academy, University of Gothenburg, Gothenburg, Sweden; 3 Geriatric Medicine, Department of Internal Medicine and Clinical Nutrition, Institute of Medicine, Sahlgrenska Academy, University of Gothenburg, Gothenburg, Sweden; 4 Department of Surgical Sciences, University of Uppsala, Uppsala, Sweden

**Keywords:** Bone mass, Osteoporosis, Physical activity, Older, Men

## Abstract

**Background:**

Detection of high-risk individuals for fractures are needed. This study assessed whether level of physical activity (PA) and a musculoskeletal composite score could be used as fracture predictive tools, and if the score could predict fractures better than areal bone mineral density (aBMD).

**Methods:**

MrOs Sweden is a prospective population-based observational study that at baseline included 3014 men aged 69–81 years. We assessed femoral neck bone mineral content (BMC), bone area, aBMD and total body lean mass by dual energy X-ray absorptiometry, calcaneal speed of sound by quantitative ultrasound and hand grip strength by a handheld dynamometer. PA was assessed by the Physical Activity Scale for the Elderly (PASE) questionnaire. We followed the participants until the date of first fracture, death or relocation (median 9.6 years). A musculoskeletal composite score was calculated as mean Z-score of the five measured traits. A Cox proportional hazards model was used to analyze the association between the musculoskeletal traits, the composite score and incident fractures (yes/no) during the follow-up period. Data are presented as hazard ratios (HR) with 95% confidence intervals (95% CI) for fracture for a + 1 standard deviation (SD) change (+ 1 Z-score) in the various musculoskeletal traits as well as the composite score. We used a linear regression model to estimate the association between level of PA, measured as PASE-score and the different musculoskeletal traits as well as the composite score.

**Results:**

A + 1 SD higher composite score was associated with an incident fracture HR of 0.61 (0.54, 0.69), however not being superior to aBMD in fracture prediction. A + 1 SD higher PASE-score was associated with both a higher composite score and lower fracture incidence (HR 0.83 (0.76, 0.90)).

**Conclusions:**

The composite score was similar to femoral neck aBMD in predicting fractures, and also low PA predicted fractures. This highlights the need of randomized controlled trials to evaluate if PA could be used as a fracture preventive strategy.

## Background

The remaining lifetime risk for fragility fractures at the age of 50 is 50% in women and 25% in men [1]. The most devastating of these, the hip fracture, is associated with excess mortality, also in comparison to other major fractures [2]. Recent studies have projected that hip fracture rates in Sweden and Denmark will increase substantially during the upcoming decades, causing not only pain, disability and death for individual patients, but also a heavy burden on the health care systems [3]. With this in mind, new preventive methods are necessary together with improved early identification of individuals at high risk for fractures. A current method of preference for identifying high risk individuals is a femoral neck areal bone mineral density (aBMD) measurement, where each standard deviation lower aBMD is associated with a two to three times higher fracture risk [4]. Another approach is to combine several risk factors, and based on these quantify the fracture risk. In FRAX, which combines clinical risk factors, with or without femoral neck aBMD, the 10-year probabilities of sustaining a hip fracture or major osteoporotic fracture are estimated [5]. However, several risk factors for fracture are not included in FRAX, such as neuromuscular function, bone quality, level of physical activity (PA) and fall risk [6–9].

Physical inactivity is a risk factor for bone mineral and muscle loss and also a risk factor for fragility fractures [9–11]. PA interventions on the other hand have been found to improve both bone mass, muscle strength [10, 12] and in children also a musculoskeletal composite score for fractures, consisting of five bone and neuromuscular traits, all associated with fracture risk [13]. The referred score may hypothetically have a better fracture predictive ability than a single trait measurement, as a fracture protecting effect may be mediated by several pathways, including muscle mass, muscle strength, bone size, bone mass and bone quality [4, 6, 14, 15]. A composite score may also be a better estimate of the PA-induced musculoskeletal benefits, as different types of PA induce different effects on the musculoskeletal system [13]. However, it remains to be shown if the composite score actually predicts fracture risk and if the score is associated with PA also in older individuals.

The primary aim of this study was, to in a cohort of older men, investigate if a musculoskeletal composite score could predict fractures, and if so, superior to the included traits as well as femoral neck aBMD. The secondary aims were to investigate if the level of PA was associated with the composite score and/or fracture incidence and if so, better than the included traits.

## Methods

The Osteoporotic Fractures in Men (MrOs) Sweden study is a prospective population-based multicenter observational study consisting of 3014 older men aged between 69 and 81 years at inclusion (mean age 75.4 ± (SD) 3.2 years), with the main aim to identify risk factors for osteoporosis and fracture. The study protocol has been described in detail previously [16]. In summary, participants were randomly selected from the Swedish national population register and invited by mail. To be eligible for the study the men had to be community-dwelling and able to walk without assistance. The participants were recruited and measured at hospitals in the three cities of Malmö (*n* = 1005), Gothenburg (*n* = 1010) and Uppsala (*n* = 999) between October 2001 and December 2004 with an attendance rate of 45% [16].

At study start, we measured height (cm) and weight (kg) using standard equipment, body mass index (BMI; kg/m^2^) was calculated as weight divided by height squared. We used dual energy X-ray absorptiometry (DXA) with femoral neck software to measure bone mineral content (BMC; g), bone area (cm^2^) and areal bone mineral density (aBMD; g/cm^2^) for the femoral neck and total body software to measure total body lean mass (kg). For the bone mass measurements, we primarily measured the right femoral neck but if this measurement was incomplete or missing we used the measurement of the left femoral neck. In Malmö and Uppsala, we used a Lunar Prodigy DXA (GE Lunar Corporation, Madison, WI, USA) and in Gothenburg a Hologic DXA Hologic QDR 4500/A-Delphi (Hologic, Inc., Bedford, MA, USA). We used quantitative ultrasound (QUS) (Hologic Sahara, Waltham, MA, USA) to measure calcaneal speed of sound (SOS; m/s) from the left foot, a measurement that has been referred to also estimating bone quality [7]. We used a Jamar® 5030 J1 hydraulic hand dynamometer (Jackson, MI, USA) with adjustable handgrip to measure hand grip strength. We performed two measurements on each hand and used the best of the four measurements. We did not measure hand grip strength if the participant had arthritis, pain in the hand or wrist or if the participant had undergone surgery in the upper extremity during the preceding 3 months.

Participants also answered a questionnaire that included questions on lifestyle, educational level, falls during the recent 12 months and medical history. We estimated physical activity with the Physical Activity Scale for the Elderly (PASE) questionnaire. This is a validated self-report questionnaire that covers different aspects of PA and activities of daily living [17]. The PASE questionnaire contains 12 different questions and renders a final score that ranges from 0 to 400, where a higher score indicates a higher level of PA. To use the data from the PASE questionnaire from an included individual we accepted a maximum of two missing values of the total 12 questions and replaced missing values by imputation of the mean score for the answered question for all participants.

The follow-up time was recorded as the date from the baseline visit until the date of first fracture, death, relocation or 31st December 2013, rendering a median follow-up time of 9.6 years. A total of 1237 (of 3014) participants died or moved during the follow-up period. Fractures during the follow-up period were identified by reviewing the archives of digital radiologic images in Malmö, Gothenburg and Uppsala, and only objectively verified fractures were included. We registered the first observation of a fracture and if a participant sustained a first fracture at multiple sites, we included only one fracture. That is, the expression fracture incidence in this study corresponds to individuals with at least one fracture.

We used IBM SPSS Statistics® version 25 for statistical analyses. We present data as absolute numbers (n) with proportions (%) or means with standard deviations (SD). For five traits that all have been reported to be associated with fracture risk (femoral neck BMC, femoral neck bone area, total body lean mass, calcaneal SOS and hand grip strength) [4, 6, 14, 15] we calculated subject specific Z-scores for each trait. The Z-scores were calculated as the number of standard deviations (SD) above or below the age-predicted value, estimated in a linear regression model with age versus included trait. The mean Z-score of the five traits were calculated as a composite score. If one or two traits were missing, we calculated the mean Z-score from the remaining traits. As we used two different types of DXA scanners all traits were transferred to Z-scores within each city cohort (Malmö, Uppsala and Gothenburg). It should also be noted that this score included hand grip strength, in contrast to the referred composite score in children which instead included knee flexion strength [13]. We used a Cox proportional hazards model to analyze the association between the musculoskeletal traits, the composite score and incident fractures (yes/no) during the follow-up period. We present hazard ratios (HR) with 95% confidence intervals (95% CI) for fracture for a + 1 SD change (+ 1 Z-score) in the musculoskeletal traits as well as the composite score. We used a linear regression model to estimate the association of level of PA, measured as PASE-score, on the musculoskeletal traits and the composite score. We regarded *p* < 0.05 as statistically significant.

## Results

We present descriptive baseline characteristics of study participants in Table 1 and incident fracture data during the follow-up period in Table 2.

**Table 1:** Baseline characteristics of study participants

	N	
Anthropometry, mean (SD)	3014	
Age (years)		75.4 (3.2)
Height (cm)		174.8 (6.5)
Weight (kg)		80.8 (12.1)
BMI (kg/m^2^)		26.4 (3.6)
Physical activity, mean (SD)	2977	
PASE-score		130.6 (61.7)
Smoker, n (%)	3011	
Never		1058 (35.1%)
Current/past		1953 (64.8%)
Alcohol, n (%)	2472	
< 2 drinks/day		2247 (74.6%)
≥ 2 drinks/day		225 (7.5%)
Medical history^a^, n (%)	3014	
Yes		1961 (65.1%)
No		1053 (34.9%)
Osteoporosis, n (%)	3006	
Yes		56 (1.9%)
No		2950 (97.9%)
Any fall during past 12 months, n (%)	3003	
Yes		495 (16.4%)
No		2508 (83.2%)
Education, n (%)	3006	
Elementary school		1397 (46.4%)
Higher education		1609 (53.4%)
Native country, n (%)	3012	
Sweden		2800 (92.9%)
Other		212 (7.0%)
Body composition (kg), mean (SD)	2950	
Total body lean mass		55.5 (6.8)
BMC (g), mean (SD)	2984	
Femoral neck		5.0 (0.9)
Bone area (cm^2^), mean (SD)	2984	
Femoral neck		5.9 (0.5)
aBMD (g/cm^2^), mean (SD)	2984	
Femoral neck		0.83 (0.13)
Quantitative ultrasound (m/s), mean (SD)	2659	
Calcaneal SOS		1551.1 (37.7)
Hand grip strength (kg), mean (SD)	2945	43.0 (7.9)

^a^History of hypertension, diabetes, coronary heart disease, congestive heart failure, chronic obstructive pulmonary disease, stroke or cancer. *N* Numbers, *%* Percentages, *BMI* Body mass index, *BMC* Bone mineral content, *aBMD* Areal bone mineral density, *SOS* Speed of sound, *SD* Standard deviation

**Table 2:** Descriptive fracture data during the follow-up period

Fracture type	Numbers
Proximal humerus	36
Collar bone	12
Wrist	48
Hand	50
Pelvis	23
Hip	153
Spine	212
Foot/ankle	42
Rib	42
Other upper extremity	18
Other lower extremity	28
Other	19
Total	683

A favorable composite score was associated with a lower fracture incidence (HR per + 1 SD = 0.61 (95% CI 0.54, 0.69)), which was similar to the corresponding HR per + 1 SD change in femoral neck BMC, aBMD and calcaneal SOS (Table 3). The model indicated that the fracture incidence was attenuated, but to a lesser extent, by each + 1 SD change in hand grip strength (HR 0.80 (95% CI 0.74, 0.87)) and total body lean mass (HR 0.91 (95% CI 0.84, 0.98). We found no association between femoral neck bone area and fracture incidence (Table 3).

**Table 3:** Hazard ratios of incident fractures during the follow-up period per + 1 standard deviation trait change

	HR	95% CI
Standard bone mass measurement		
Femoral neck aBMD	0.62	0.57, 0.67
Traits included in the composite score		
Femoral neck area	1.04	0.96, 1.12
Femoral neck BMC	0.67	0.62, 0.73
Calcaneal SOS	0.68	0.62, 0.75
Hand grip strength	0.80	0.74, 0.87
Total body lean mass	0.91	0.84, 0.98
Composite score		
Composite score	0.61	0.54, 0.69
PASE		
PASE-score	0.83	0.76, 0.90

*HR* Hazard ratio, *95% CI* 95% confidence interval, *BMC* Bone mineral content, *aBMD* Areal bone mineral density, *SOS* Speed of sound, *PASE* Physical Activity Scale for the Elderly

A better PASE-score was associated with a favorable composite score and a better value in each of the included individual traits, except femoral neck bone area (Table 4). Moreover, each + 1 SD change in PASE-score was associated with a lower fracture incidence (HR 0.83 (95% CI 0.76, 0.90)).

**Table 4:** Linear regression model examining the change in trait Z-scores per + 1 standard deviation in PASE-score

	Individuals (n)	β	S.E	*P* value	95% CI
Standard bone mass measurement					
Femoral neck aBMD	2955	0.09	0.02	< 0.001	0.05, 0.12
Traits included in the composite score					
Femoral neck area	2955	0.02	0.02	0.24	−0.01, 0.06
Femoral neck BMC	2955	0.09	0.02	< 0.001	0.05, 0.12
Calcaneal SOS	2630	0.07	0.02	< 0.001	0.03, 0.11
Hand grip strength	2914	0.20	0.02	< 0.001	0.16, 0.24
Total body lean mass	2917	0.09	0.02	< 0.001	0.06, 0.13
Composite score					
Composite score	2977	0.09	0.01	< 0.001	0.07, 0.12

*N* Numbers, *β* Linear regression coefficient, *S.E* Standard error, *95% CI* 95% confidence interval, *BMC* Bone mineral content, *aBMD* Areal bone mineral density, *SOS* Speed of sound, *PASE* Physical Activity Scale for the Elderly

## Discussion

In this prospective, population-based cohort study of older men we found that a musculoskeletal composite score predicts incident fractures similarly to a femoral neck aBMD measurement. We also found that the composite score had a positive dose-response relationship with PA and that a higher PASE-score was associated with a lower fracture incidence.

Our data support publications that infer that each of the traits aBMD, BMC, calcaneal SOS, muscle mass and hand grip strength are useful for fracture prediction [4, 6, 9, 18, 19]. The composite score was, as we hypothesized, also a good predictive estimate for fractures, however, similar compared to the measurements of femoral neck aBMD, femoral neck BMC and calcaneal SOS. Furthermore, we found that total body lean mass was a statistically significant predictor for fracture, although with a somewhat weaker point estimate than femoral neck aBMD, femoral neck BMC, calcaneal SOS and the composite score. As lean mass reflects BMI, which is associated to fracture risk [20], this finding was expected.

Since the composite score was similar to aBMD in predicting fractures, we cannot recommend to use it in fracture risk assessments in older men. The reason is that the measurements by three different techniques to calculate Z-scores of five different traits would take more time without any obvious improvement in fracture prediction.

Even if not being the main aim of this study, it is also of interest to register that more of the included single traits were significant predictors of fractures, such as hand grip strength. This method has previously been recommended as a fracture predictive tool since it is inexpensive and possible to conduct in all health care units without advanced, bulky or costly equipment [6, 8]. However, it should be emphasized that the predictive ability in old men seems to be inferior to that of both DXA, calcaneal QUS and the composite score. Since femoral neck aBMD, calcaneal QUS and the composite score all seem to have a better fracture predictive ability compared to hand grip strength, it seems questionable if hand grip strength in old men should be recommended as a fracture predictive tool.

Large bone size has in many studies been associated with low fracture risk [21, 22]. This view is supported by mechanical calculations, as the strength of a tubular bone increases with the fourth power of the radius [21]. We could however not identify femoral neck bone area as a statistically significant predictor for fracture in the old men. It is known that bone size increases with age as a result of both increased medullary and periosteal diameter [21]. However, if the medullary expansion is greater than the periosteal expansion, this results in a thinner cortical shell with a lower capacity to withstand external forces. The ratio between the diameter of the bone (estimated as the maximum distance between the center of mass and the outer cortex) and cortical shell thickness is sometimes called buckling ratio [23]. A high buckling ratio as well as a wide and large femoral neck bone have in old individuals been reported to be associated with an increased risk for hip fracture [24–26]. That is, bone size may have a different impact on fracture risk in young and old individuals. We therefore speculate that bone size should always be related to the cortical shell thickness and intracortical porosity for estimations of a bone’s resistance to trauma.

In a previous study, we speculated that a composite score may possibly capture the PA-induced musculoskeletal effects better than a single trait measurement [13]. In the current study we found that this does not seem to be the case, at least not in older men, since the association between the composite score and PA in this study was similar to that of PA and the bone mass traits and lean mass. The association between hand grip strength and PA was even stronger than for the other traits, including the composite score. Our study therefore supports publications that infer that muscle strength in older men seems to be associated with a reduced fracture risk which could be the result of higher PA [19, 27]. However, we must emphasize that increased PA was positively associated also with bone mass although to a lesser extent than muscle strength [19, 28, 29]. Improving muscle mass and neuromuscular function by increased PA in older individuals should therefore be considered as an important task, since this could reduce falls and thereby also fractures [19, 27]. Some researchers even infer that we in older individuals should shift focus from trying to improve bone mass to instead trying to improve neuromuscular function [30].

Strengths of this study include the large sample size and the population-based study design with a relatively long follow-up period. In addition, the collection of both bone and muscle measurements as well as evaluation of PA by a validated questionnaire and fractures by objective verification are further strengths. Study limitations include a participation rate below 50% and the extensive baseline exam that may have increased the risk for selection bias as the frailest men may have declined participation. Another limitation is that the questionnaires relied on self-report data, which could result in recall bias. Also, the cohort consisted almost entirely of white older men, which limits inferences to this group.

## Conclusions

In summary, this study shows that a musculoskeletal composite score which includes five musculoskeletal traits, seems to be equally useful (but not better) in predicting fractures in old men as a femoral neck aBMD. We also found that a higher level of PA was associated with a lower fracture incidence, and with a beneficial composite score. The findings highlight the need of randomized controlled trials to evaluate if PA could be used as a fracture preventive strategy.

## Abbreviations

95% CI: 95% confidence interval
aBMD: Areal bone mineral density
BMC: Bone mineral content
BMI: Body mass index
DXA: Dual energy X-ray absorptiometry
HR: Hazard ratio
PA: Physical activity
PASE: Physical Activity Scale for the Elderly
QUS: Quantitative ultrasound
SD: Standard deviation
SOS: Speed of sound
TBLM: Total body lean mass
